# Application of factor analytic and spatial mixed models for the analysis of multi-environment trials in common bean (*Phaseolus vulgaris* L.) in Ethiopia

**DOI:** 10.1371/journal.pone.0301534

**Published:** 2024-04-18

**Authors:** Tarekegn Argaw, Brehanu Amsalu Fenta, Ermias Assefa

**Affiliations:** 1 Climate and Computational Science Research Directorate, Ethiopian Institute of Agricultural Research (EIAR), Addis Ababa, Ethiopia; 2 Melkassa Agricultural Research Center, Ethiopian Institute of Agricultural Research (EIAR), Adama, Ethiopia; 3 Bioinformatics & Genomics Research Directorate, Bio and Emerging Technology Institute (BETin), Addis Ababa, Ethiopia; Universidade Federal de Minas Gerais, BRAZIL

## Abstract

Common bean (*Phaseolus vulgaris* L.) is one of the most important grain legumes consumed globally, especially in Ethiopia, for its edible seeds, cash crops, and supply of protein for farmers. Efficient statistical methods must be employed for the evaluation of common bean varieties to accurately select superior varieties that contribute to agricultural productivity. The objective of this study was to identify promising large mottled bean varieties through analysis of multi-environment trials (MET) data using multiplicative spatial mixed models. In this study, 16–18 large mottled common bean varieties, including one check, were sown across nine growing environments in Ethiopia using lattice and alpha lattice designs, with three replications laid out in a square or rectangular (row by column) array of plots, respectively during the main cropping season from 2015 to 2018. We present a linear mixed model analysis that integrates spatial and factor analytic (FA) models, and the heritability measure was used to evaluate the efficiency of these models with the conventional analysis. The analysis of the spatial model, and more significantly, the spatial+FA model, revealed a notable enhancement in heritability. With the exception of a trial conducted at Kobo, a genotype DAP 292, found to be good performing for days to flowering and maturity, but for yield only across four clusters of trials, C2, C3, C5 and C7, formed with trials of relatively high genetic variance. Across these clusters, the yield advantage of this variety over the check ranged from 10–32%. This genotype also has a yield that is somewhat comparable to the check across the remaining clusters. Overall, both the spatial and factor analytic models proved to be effective approaches for analyzing the data in this study. The analysis of multi-environment trial data through the use of more efficient statistical models can provide a more robust platform for evaluating common bean varieties with greater confidence in selecting superior varieties across a range of environments. Hence, scaling up the use of this efficient analysis method is indispensable for enhancing the selection of superior varieties.

## Introduction

Common bean (*Phaseolus vulgaris* L.) is one of the most important grain legumes grown on over—half a million hectares annually in Ethiopia during the main and short (belg) growing seasons, serving as a nutritious source of dietary protein for rural and urban communities [1]. It is the most important pulse, providing various bean products (green pods, fresh seed, as well as dry seed) and is popular as it offers a staggered and prolonged food supply. It also has health benefits, being rich in protein and providing a good source of iron and zinc, which plays a role in mental development [2, 3]. Aside from contributing to food and nutritional security, common bean provides a consistent and inclusive source of income for smallholder farmers and value chain actors involved in this sector [4, 5]. It is also one of the important commodities generating more than 100 million USD foreign exchange earnings for the country every year [6, 7]. It is an early-maturing crop and can be easily intercropped with other cereals like maize and sorghum, and hence serves as a key component in intensifying production in smallholder farming systems [8]. Its ability to fix nitrogen also helps as a good rotation crop and contributes to soil fertility improvement. In a nutshell, the versatility of the common bean crop and its contribution to a household’s food, income, diet, health and even environmental security is remarkable, and it is one of the strategic crops for the lowland agro-ecologies of the country [1].

The evaluation of crop varieties through field experimentation is the main activity in plant breeding research to identify superior varieties that contribute to agricultural productivity. Superior crop varieties are selected through one of the two statistical approaches: either by determining the difference between specific pairs of varieties or by ranking the estimated genotypic effect [9]. The validity of the experimental design and the statistical model used during data analysis greatly influence the accuracy of variety evaluation. Any incorrect evaluation can be attributed to potential flaws in these approaches, ultimately impacting the reliability of the developed variety [10].

The classical method frequently used for the analysis of multi-environment trial (MET) data sets is analysis of variance (ANOVA), which is computed using ordinary linear models (LMs) that make use of ordinary least square (OLS) methods for the estimation for unknown parameters, and an ANOVA table for source of variation test with overall f-test and post hoc multiple comparison test with mostly least significance difference (LSD) and duncan test for observed means. Further analysis is also conducted for more information on the genotype by environment interaction (GEI) component through multiplicative analysis techniques, such as AMMI (additive main effects and multiplicative interaction) and GGE (genotype plus genotype by environment interaction) techniques [11, 12]. As pointed out by several authors [13, 14], a major drawback of AMMI and GGE models is that they are unable to handle error variance heterogeneity across trials, spatial variation within trials, unbalanced data, and missing values.

The linear mixed model (LMM), an extended LM (linear model), can account for the spatial variability in the experimental units through incorporating fixed and random terms in the model for systematic variability and through relaxing the distributional assumptions surrounding the residual error [14, 15]. Yang [16] explains that the Linear Mixed Model (LMM) is powerful and flexible statistical model, employing Best Linear Unbiased Prediction (BLUP) to estimate random effects and obtain unbiased estimates of variance components using Restricted Maximum Likelihood Estimation (REML). LMMs are suitable for all types of field trial data (balanced and unbalanced), and to go for extended analysis using spatial and FA (factor analytic) models [17]. MET data analysis under LMM with random genotype could improve precision for genotype ranking by shrinking the estimates of genotype effects towards their true value. More improvements have been made in modeling spatial variation within trials, popularized by the approach of Gilmour et al. [18], focusing on three scales: global, extraneous, and local trends; and modeling the covariance structure of GEI effects through FA models.

While common bean offers numerous benefits, it also presents certain challenges such as biotic and abiotic stress, a scarcity of released varieties, particularly mottled beans, and limited technology multiplication and promotion. Notably, despite these challenges, Ethiopian farmers held a strong preference for the culinary characteristics of the large mottled common bean varieties. These beans were highly valued for their ability to swell during the cooking process, resulting in an aesthetically pleasing dish with a delightful and flavorful taste [19]. Thus, this study was planned to generate a high-yielding and stable large mottled bean variety that might suit the local and regional market through data analysis of multi-environment trials using multiplicative spatial mixed models (using spatial and FA models under the LMM frame work).

## Material and methods

### Plant material and experimental design

A total of eighteen large seeded mottled common bean genotypes including seventeen introduced lines sourced from the International Centre for Tropical Agriculture (CIAT) and one check were used in this study (Table 1). Among the total number of trials conducted, eleven trials were laid out using triple lattice design with sixteen entries, while the remaining five trials were designed by alpha lattice with eighteen entries and three replications (Table 3). These trials were laid out in a rectangular (square) array of plots, arranged in rows and columns. Each plot was consisting of six planting rows of 4 m long with 0.4 m spacing between rows and 0.1m between plants. The central four rows were harvested for grain yield from each plot. Fertilizer was applied to each plot at the rate of 18 kg N and 46 kg P_2_O_5_ ha^-1^ in the form of di-ammonium phosphate (DAP) at planting. All other management practices were uniformly applied for each trial.

**Table 1:** List of genotypes used in the study.

Entry	Genotype	Pedigree	Seed source
1	DAB 292	CAL96/SAB621/SAB686	CIAT
2	DAB 247	SAB628/CAL143//SAB659	CIAT
3	DAB 288	KATB1/SAB618//SAB659	CIAT
4	DAB 251	SAB628/CAL143//SAB659	CIAT
5	DAB 237	SAB628/CAL143//SAB686	CIAT
6	DAB 220	SAB628/CAL143//SAB659	CIAT
7	DAB 320	G23227/SAB627//SAB659	CIAT
8	DAB 396	CAL143/SAB620//SAB626	CIAT
9	NUA 94	CAL143//CAL143/G14519	CIAT
10	DAB 298	CAL96/SAB621/SAB686	CIAT
11	DAB 283	KATB1/SAB618/SAB659	CIAT
12	NUA 99	CAL143//CAL143/G14519	CIAT
13	DAB 245	SAB628/CAL143//SAB659	CIAT
14	DAB 278	SAB628/CAL143//SAB659	CIAT
15	GLP 2 (Check)	G8043	EIAR/MARC
16	DAB 265	SAB628/CAL143//SAB659	CIAT
17	DAB 259	SAB628/CAL143//SAB659	CIAT
18	DAB 241	SAB628/CAL143//SAB659	CIAT

MARC: Melkassa Agricultural Research Center

### Description of experimental sites

The experiment was conducted in nine common bean growing areas, namely, Areka, Arsinegelle, Alemtena, Gofa, Melkassa, Kobo, Pawe, Sekota, and Sirinka, Ethiopia during the 2015–2018 main cropping seasons. These nine locations represent the different common bean growing agro-ecologies of Ethiopia, and detailed descriptions of the study locations are presented in Table 2.

**Table 2:** Detailed agro-ecological and weather descriptions of the study locations.

Location	Code	Soil type	Altitude (m.a.s.l)	Latitude	Longitude	Annual averages		
						Min (°c)	Max (°c)	Rainfall (mm)
Areka	AK	Alisol	1780	7° 06’N	37°69’E	14.2	26.2	1438
Arsinegele	AN	Nitosols	1890	7° 35’N	38°65’E	11.1	25.2	876
Alemtena	AT	Andosols	1610	8°18’N	38°57’E	12.9	29.8	728
Gofa	GF	Acrisols	1297	6°36’N	37°12’E	29.4	17.6	1338
Melkassa	MK	Andosols	1550	8° 30’N	39° 21’E	16.0	28.8	763
Kobo	KB	Nitosols	1468	12°09′N	39°38′E	18.0	27.0	615
Pawe	PW	Nitosols	1120	11°19’N	36°24’E	16.3	32.6	1587
Sekota	SK	Vertisol	1850	12°14′ N	38°30’E	12.9	32.9	789
Sirinka	SR	Eutricvertiol	1880	11°08’N	39°28’E	15.3	28.3	806

m.a.s.l = meters above sea level, E = east, N = north, Min = minimum, Max = maximum: Source: Melkassa Agricultural Research Centers and National Meteorology Agency

### Data collected

In this study, two types of data, namely plot-based and plant-based data, were collected. Data collected on plot basis includes days to flowering, days to maturity, and grain yield, while the number of pods per plant data was collected on plant basis. The description of the collected data/traits has been shown as follows:

- Days to flowering (DTF): This was measured as the number of days from planting to when 50% of the plants in a plot had at least one open flower.
- Days to physiological maturity (DTM): the number of days from planting to when at least 90% of the pods on 85% of the plants in the plot turned yellow.
- Grain yield per plot (YLD): Before the seed yield was measured, seed moisture content (MC) was determined using a digital moisture tester and finally used to calculate the adjusted yield.
- Adjusted grain yield (YLD_adj_) or **s**eed yield data from central four rows of the plot were adjusted to 12.5 seed Moisture Content (MC) using the equation [20]
$$YLDadj=\left[\left(\left(100-MC\right)/\left(100-12.5\right)\right)\;*\;YLD\right]$$
  And the adjusted grain yield is expressed in kilogram per hectare, or in tons per hectare for the analysis result presentations.
- Number of pods per plant (NPP): This was measured from five randomly selected plants in the harvestable rows and average number of mature pods counted at harvest.

Table 3 shows sixteen trials designated using the location code listed in Table 2, the last two digits of the trial year and the first letter of the name of the trial stage, PVT and NVT, which stand for preliminary variety trial and national variety trial, respectively. Table 3 also shows the number of replications (Rep) and the dimension of each trial (row and column), as well as the average mean value of the measurements of each trait across trials. The trait grain yield (YLD), which we use throughout the analysis, is the harvested grain yield expressed in tons per hectare. The concurrence of entries between trials, both within and between years, was high, with a minimum of sixteen entries occurring in common within and between years. In this study, trial and environment are used interchangeably, where environment/trial is year by location combination.

**Table 3:** Summary of trial parameters and trait measurements across trials.

Trials	Trial parameters				Average measurement			
	Rep	Row	Column	Entry	YLD	PPP	DTF	DTM
AK16P	3	12	4	16	1.07	6.80	35.9	74.88
AN15P	3	12	4	16	2.48	12.52	47.38	90.60
AN17N	3	12	4	16	3.70	10.94	31.96	88.52
AN18N	3	9	6	18	2.10	9.97	44.15	90.06
AT16P	3	12	4	16	2.21	11.01	36.02	74.40
AT17N	3	12	4	16	2.84	10.64	29.9	70.88
GF17N	3	12	4	16	1.62	19.83	30.88	68.50
GF18N	3	9	6	18	0.93	11.99	32.54	82.19
KB17N	3	12	4	16	1.85	6.50	44.77	71.21
MK16P	3	12	4	16	2.44	11.28	30.88	65.94
MK17N	3	12	4	16	3.46	13.86	28.48	73.38
MK18N	3	9	6	18	1.92	11.50	35.63	79.41
PW16P	3	12	4	16	1.78	6.54	38.02	77.29
PW17N	3	12	4	16	0.79	15.95	33.02	76.46
SK18N	3	9	6	18	1.34	10.46	42.28	78.70
SR18N	3	9	6	18	2.14	10.12	38.89	80.33

YLD: grain yield; PPP: pod per plant; DTF: day to flowering; DTM: day to maturity

### Statistical models

#### Linear mixed model

Consider a MET dataset collected from *t* trials (environments can be used instead) in which *m* varieties are grown (all varieties may not be grown in all trials). The *j*^*th*^ trial, *j* = 1…*t*, consists of *n*_*j*_ plots arranged in a rectangular array with *c*_*j*_ columns by *r*_*j*_ rows (*n*_*j*_ = *c*_*j*_*r*_*j*_). Let ***y***_*j*_ be the (*n*_*j*_ x 1) data vector for trial *j*, ordered as rows within columns, and let $y=(y_{1}^{\prime },y_{2}^{\prime },\ldots ,y_{t}^{\prime })\prime$ be the (*n* x 1) data vector combined across the *t* trials, where $n=\sum_{j=1}^{t}n_{j}$. The linear mixed model for y can then be written as

$$y=X\alpha +Z_{g}\gamma_{g}+Z_{p}\gamma_{p}+\varepsilon$$ (1)

where *α* is vector of fixed effects (including terms for the grand mean, the environment’s main effects, global spatial trends at each trial, and other trial-specific fixed effects) with an associated design matrix *X* (assumed to be full column rank), *γ*_*g*_ is the *mt* x 1 vector of random genetic (or variety by trial) effects with associated design matrix *Z*_*g*_, *γ*_*p*_ is a vector of non-genetic (or peripheral) random effects (including terms associated with the blocking structure at each trial, and other trial-specific random effects), with associated design matrix *Z*_*p*_, and *ε* is the n x 1 vector of residual errors across all trials.

The random effects from the linear mixed model (Eq 1) are assumed to follow a normal distribution with mean zero vector and variance-covariance matrix, that is

$$E\left(\begin{array}{c} \gamma_{g}\\ \gamma_{p}\\ \varepsilon \end{array}\right)=\left(\begin{array}{c} 0\\ 0\\ 0 \end{array}\right)‘\;\text{var}\left(\begin{array}{c} \gamma_{g}\\ \gamma_{p}\\ \varepsilon \end{array}\right)=\left[\begin{array}{ccc} G_{g} & 0 & 0\\ 0 & G_{p} & 0\\ 0 & 0 & R \end{array}\right]$$

where *G*_*g*_ is variance matrix for genetic effects, *G*_*p*_ is the variance matrix of non-genetic (or peripheral) random effects and *R* is the variance matrix of the random error effects.

### Model for genetic effects (*γ*_*g*_)

Smith et al. [21] presented an alternative parsimonious model for *γ*_*g*_ using a factor analysis approach to provide a variance structure for the genetic variance matrix *G*_*g*_. This model can adequately represent the nature of heterogeneous variances and covariance occurring in most MET data. Thus, the *γ*_*g*_ can be modeled with multiplicative terms. That is

$$\begin{array}{c} \gamma_{g}=(\lambda_{1}⊗I_{m})f_{1}+\ldots +(\lambda_{k}⊗I_{m})f_{k}+\zeta \\ \;=(\Lambda ⊗I_{m})f+\zeta \end{array}$$ (2)

where *λ*_*r*_ is the *t* × 1 vector of loadings, *f*_*r*_ is the *m* × 1 vector of factor scores (*r* = 1…*k*), *ζ* is the *mt* × 1 vector of residuals, Λ is the *t* × *k* matrix of loadings {*λ*_1_ … *λ*_*k*_} and *f* is the *mk* × 1 vector of factor scores $(f_{1}^{\prime }f_{1}^{\prime }\ldots f_{k}^{\prime })\prime$. The random effects *f* and *ζ* are assumed to follow a normal distribution with zero mean vector and variance-covariance matrix

$$\left[\begin{array}{cc} G_{f}⊗I_{m} & 0\\ 0 & \Psi ⊗I_{m} \end{array}\right]$$

where Ψ is a diagonal matrix of specific variances represents the residual variance not explained by the factor model, that is Ψ = *diag* (Ψ_1_ … Ψ_t_). The factor scores are commonly assumed to be independent and scaled to have unit variance, so that *G*_*f*_ = *I*_*k*_.

The genetic effects *γ*_*g*_ can be considered as a two dimensional (genotype by environment) array of random effects, and can be assumed to have a separable variance structure for the (*mt × mt*) variance matrix *G*_*g*_ that can be written as

$$G_{g}={\text{G}}_{\text{e}}⊗G_{v}$$

where *G*_*e*_ is the *t* × *t* genetic variance matrix representing the variances at each trial and covariances between trials, and *G*_*v*_ is the *m* × *m* symmetric positive definite matrix represents variances of environment effects at each genotype and the covariances of environment effects between genotypes. It is typically assumed that the varieties are independent and that *G*_*v*_ = *I*_*m*_. However, if the pedigree information of the varieties is available, other forms of *G*_*v*_ can be applicable [22, 23]. Based on Eq 2 the variance of genetic effects would be

$$\begin{array}{c} \text{var}(\gamma_{g})=(\Lambda \Lambda \prime +\Psi )⊗I_{m}\\ \;={\text{G}}_{\text{e}}⊗I_{m} \end{array}$$

Thus, the FA model approach results in the following form for *G*_*e*_

$$G_{e}=\Lambda \Lambda \prime +\Psi$$

In the model, the variance parametric in these variance matrices are directly estimated using REML estimation method.

### Model for non-genetic effects (*γ*_*p*_)

The random non-genetic effects *γ*_*p*_ can be considered as sub- vectors ${\gamma_{pj}}^{\left(b_{j}\times 1\right)}$ for each trial, where *b*_*j*_ is the number of random terms for trial *j*. These random terms are based on terms for blocking structure (replicate blocks or rows and columns of the field). In the analysis of MET data, the sub-vectors of *γ*_*p*_ are typically assumed to be mutually independent, with variance matrix *G*_*pj*_ for trial j, with the block diagonal form given below. Thus, there is a variance matrix for the set of none-genetic effects at each trial, That is,

$$G_{p}={{⊕}^{t}}_{j}G_{pj}=\left[\begin{array}{cccc} G_{p1} & 0 & \cdots & 0\\ 0 & G_{p2} & \cdots & 0\\ \vdots & \vdots & \ddots & \vdots \\ 0 & 0 & \cdots & G_{pt} \end{array}\right]$$

The most common form for the variance matrix of these extraneous effects is a simple variance component structure, where $G_{pj}=\sigma_{j}^{2}I_{bj}$.

### Model for residual effects (*ε*)

In the analysis of MET data using a linear mixed model, the vector for residual effects *ε* can be partitioned into residual effects within each individual trial. That is, $\varepsilon =(\varepsilon_{1}^{\prime }\ldots .\varepsilon_{t}^{\prime })\prime$ where *ε*_*j*_ is the *n*_*j*_ × 1 vector of residual effects for the j^th^ trial. The variance matrix for the residual effects is assumed to be $R={⊕}_{j=1}^{t}R_{j}$ where *R*_*j*_ is the variance matrix for the j^th^ trial. Individual trial residual effects can be analyzed employing spatial methods of analysis that account for local or plot-to-plot variation. Each *R*_*j*_ in this case will have its own spatial covariance structure [18]. Variety trials that have row by column arrangement and ordered as rows within columns allow separable spatial models of the form.

$$R_{j}=\sigma_{j}^{2}\Sigma_{c_{j}}(\rho_{c_{j}})⊗\Sigma_{r_{j}}(\rho_{r_{j}})$$

where $\Sigma_{c_{j}}$ and $\Sigma_{r_{j}}$ represent spatial correlation structures with parameters in $\rho_{c_{j}}$ and $\rho_{r_{j}}$ for the column and row directions, respectively. In both the row and column directions, we typically use an autoregressive spatial structure of order one, with $\rho_{c_{j}}$ and $\rho_{r_{j}}$ each containing a single autocorrelation parameter.

For spatial auto-correlation in the row direction only, the model simplifies to $R_{j}=\sigma_{j}^{2}I_{n_{cj}}⊗\Sigma_{r_{j}}(\rho_{r_{j}})$, where *n*_*cj*_ is the number of columns for trial j. Similarly, $R_{j}=\sigma_{j}^{2}\Sigma_{c_{j}}(\rho_{c_{j}})⊗I_{n_{rj}}$ would be the reduced form for spatial auto-correlation in the column direction only, where *n*_*rj*_ is the number of rows for trial j. We can have also no spatial covariance in either direction. Thus, the model simplified to an IID variance structure of the form $R_{j}=\sigma_{j}^{2}I_{n_{cj}}⊗I_{n_{cj}}=\sigma_{j}^{2}I_{n_{j}}$.

In this study, we conducted MET data analysis that integrated both spatial models for residual and FA for genetic effects under a linear mixed model. And this is referred to interchangeably as multiplicative spatial mixed model analysis or spatial+FA model analysis throughout the paper.

### Heritability formula

Following the approach of Cullis et al. [24], the heritability ($H_{j}^{2}$) value for the j^th^ trial can be calculated from a generalized formula that takes unbalanced data into account, as

$$H_{j}^{2}=1-\frac{A_{j}}{2{\sigma_{gj}}^{2}}$$ (3)

where *A*_*j*_ is the average pairwise prediction error variance of genetic effects for the *j*^*th*^ environment and ${\sigma_{gj}}^{2}$ is the genetic variance at environment *j*

### Statistical inference procedures and software

Estimation in the linear mixed model involves determining the values of fixed and random effects, *α*, *γ*_*g*_ and *γ*_*p*_, as well as the variance-covariance parameters in *G*_*g*_, *G*_*p*_ and *R*. This estimation process consists of two interconnected steps. Firstly, the variance parameters of the model are estimated using a REML, which was introduced by Patterson and Thompson in [25]. Secondly, the fixed and random effects are estimated using two different techniques: best linear unbiased estimation (BLUE) for the fixed effects and best linear unbiased prediction (BLUP) for the random effects. To assess the significance of the random effects in the linear mixed model, the Residual Maximum Likelihood Ratio Test (REMLRT) can be employed. However, it is important to note that the REMLRT is only applicable when comparing the fit of two nested models that share the same fixed effects. On the other hand, the significance of the fixed effects in a linear mixed model can be determined using the Wald test. The classic Wald statistic follows an asymptotic chi-squared distribution. However, this test is sometimes regarded as anti-conservative, according to Butler et al. [26]. To address this issue, Kenward and Roger [27] introduced an adjusted Wald statistic and an approximation based on the F distribution, which demonstrated good performance across various scenarios. The licensed ASReml-R was used to fit all models in this study [26].

In the first step, separate analysis for each trial was undertaken to account for non-genetic effects through spatial models using the approach of Gilmour et al. [18]. Model selection was implemented after specifying the base model (specified with random replication and genotype effect with an id (row) x id (column) variance structure for residual). The first choice of spatial model was a separable autoregressive process in the field row and column directions for local trend at each trial. However, some trials were specified with an autoregressive process only in the row direction (these trials did not have enough column size). With the help of model diagnosis, in particular the sample variogram [18], terms were then added to the model to accommodate the global and extraneous variation (both systematic and random) associated with the row and column direction. The significance of adding terms is also checked through statistical tests (the wald test for fixed terms and the REML likelihood ratio test for random terms). Once the global and extraneous variation were modeled through significant added terms, the specified separable autoregressive process in modeling local variation at each trial was also assessed through the REML likelihood ratio test. And only terms that showed a significant effect were retained in the model.

The second step analysis, the analysis of genetic effects that includes GEI effects, was carried out using the model fitting procedures demonstrated by Kelly et al. [15]. Thus, the first genotype by environment model implemented in the second step analysis was a combined form of individual trial models, constructed in step one analysis, using the *diag* function built into the Asreml-R package. The model forms the basis for a sequence of models to fit into the MET data analysis. It helps us to organize the trial-specific models in combined form, and to confirm the presence of genetic variance in each trial. If any trial is found to have no genetic variance, then it would be excluded from the MET data analysis. There might also be fixed terms found to be of non-significance in the model and forced to be dropped from the models.

## Results

### Modeling non-genetic variation

**Fig 1:**
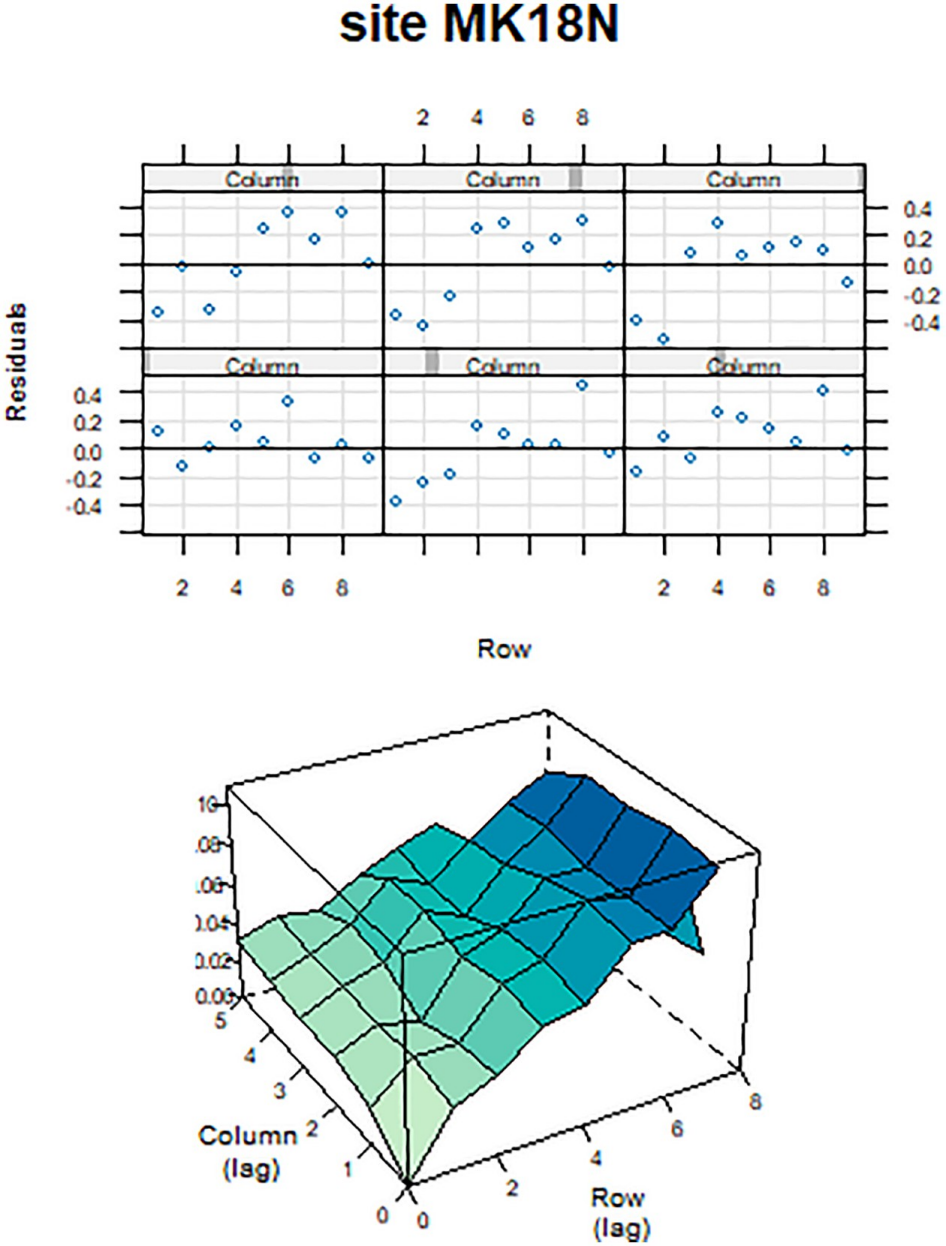
Trellis and sample variogram plot of the residuals from a base model for yield at trial MK18N.

A typical model diagnostic plot (Trial MK18N for YLD) from the Metplot package, which is a part of ASReml-R, is depicted in Fig 1. The plots show the presence of a global linear trend and extraneous variation across the rows of the field of the trial. These variations were accommodated in the model by including fixed linear and random terms, denoted by the model terms, lrow and row, respectively, and their significance was tested through the right tests. Following the same approach, appropriate spatial analysis was done for each trait and for all trials to model the none-genetic variation by including terms for non-genetic effects in the base model.

### Modeling genetic variation

In this study, we found some trials had no genetic variance (no genetic variance at MK16P for YLD, at AK16P, KB17N, MK16P, GF18N and SR18N for PPP, and at GF17N, MK17N and SR18N for DTM). These were excluded from the subsequent analysis.

Following that, FA models were considered while keeping the spatial models provided in the *diag* model. This was simply done by replacing the *dig* function with the *fa* function of Asreml-R. The adequacy of the FA models of several k orders was formally tested as it fitted within a mixed model framework. The model with k factors, denoted FA-k, is nested within the model with k + 1 factor. REMLRT was used to compare such models. Table 4 presents the results for the comparison of the FAs models for each trait. It contains the total percentage of the genotype by environment (GxE) variance (%var) that was explained by the factor components of the model, residual log-likelihoods (LR), and tests (REMLRT). Model comparisons for YLD were done up until FA-5. Significance improvement was observed in those comparisons, except for the comparison of FA-4 with FA-5 at a 5% level of significance, and the model at FA-4 was taken to be the final model for YLD. Similarly, FA model comparison was made for the rest of the traits, and as a result, FA-1 was found to be the final parsimonious model for PPP and FA-3 for DTF and DTM. The final FA models, except for YLD, provide a satisfactory fit for almost all trials. For YLD, we found that an FA-4 provides insufficient fits in some trials. This shows that these trials were generally not as well correlated with the other sites [28].

**Table 4:** FAs model comparison through total percentage of the GxE variance (%var), Residual log-likelihoods (LR), and Residual Maximum Likelihood Ratio Tests (REMLRT) for each trait.

Traits	FAs Models	%var	LR	REMLRT	Final model
YLD	FA-1	17.7	552.8	-	FA-4
	FA-2	28.6	563.8	0.039	
	FA-3	62.2	576.7	<0.001	
	FA-4	68.5	588.9	0.011	
	FA-5	82.2	597.1	0.059	
PPP	FA-1	83.6	-769.2	-	FA-1
	FA-2	93.0	763.2	0.101	
DTF	FA-1	88.5	-629.6	-	FA-3
	FA-2	92.2	-615.3	0.003	
	FA-3	98.0	-600.5	0.003	
	FA-4	98.9	-595.5	0.532	
DTM	FA-1	75.5	-696.5	-	FA-3
	FA-2	87.3	-688.9	0.125	
	FA-3	95.7	-679.7	0.005	
	FA-4	98.4	-675.1	0.328	

YLD: grain yield; PPP: pod per plant; DTF: day to flowering; DTM: day to maturity

### Comparison of conventional, spatial, and spatial+FA analysis

**Fig 2:**
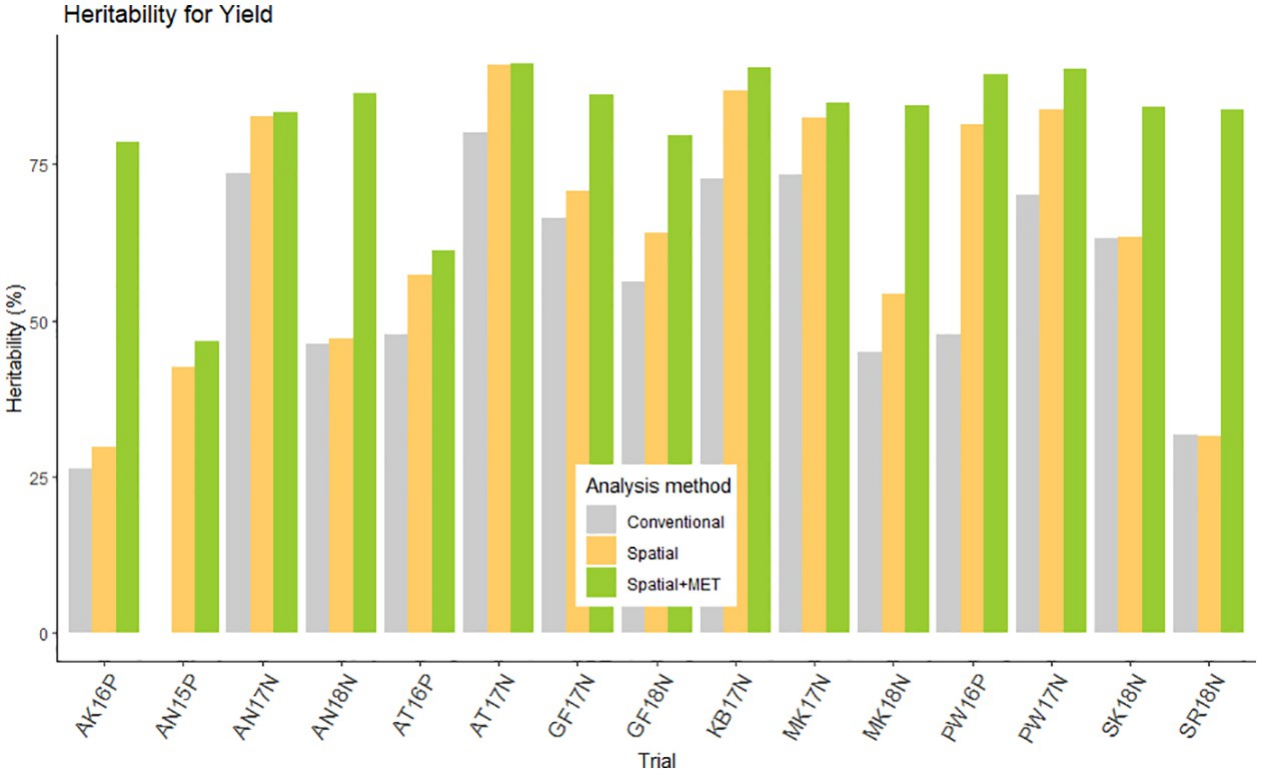
Heritability of yield for each trial using three different method of data analysis (conventional, spatial and spatial+FA).

In this study, we examined three statistical methods of MET data analysis in terms of heritability measures for the precision and accuracy of evaluating the performance of common bean varieties. Fig 2 presents the heritability of YLD at each trial using conversional, spatial, and spatial+FA analysis. It shows heritability improvement when we use spatial analysis, and more improvement resulted from spatial+FA analysis.

### The Spatial+FA analysis

The genetic variance (Gvar), error variance (Evar), and heritability (H^2^) for each trial and trait from the final fitted Spatial+FA models are presented in Table 5. Empty cells in the table show those trials excluded from the analysis due to zero genetic variance. The genetic variances range from 0.01 to 0.15 for yield (YLD), from 0.50 to 11.83 for pod per plant (PPP), from 0.02 to 8.63 for day to flowering (DTF), and from 0.06 to 5.33 for day to maturity (DTM). The heritability measure for YLD across trials ranges from 61 to 91%, with a notable exception of trial AN15P, which has 46% heritability. We found high heritability measures for the DTF and DTM, ranging from 82 to 99% and 77 to 96%, respectively. In addition, heritability measures for PPP ranged from 64 to 90% across trials.

**Table 5:** Genetic variance (Gvar), error variance (Evar), and heritability (H^2^) for each trial from the final fitted Spatial+FA models analysis for grain yield (YLD), pod per plant (PPP), day to flowering (DTF), and day to maturity (DTM).

Trial	DTF			YLD			DTM			PPP		
	Gvar	Evar	H^2^	Gvar	Evar	H^2^	Gvar	Evar	H^2^	Gvar	Evar	H^2^
AK16P	2.8	5.82	95.16	0.009	0.04	78.54	3.54	1.06	93.87	-	-	-
AN15P	1.62	1.75	82.45	0.021	0.156	46.58	1.32	3.64	96.17	4.641	8.087	90.51
AN17N	6.13	2.2	97.63	0.087	0.055	83.24	4.67	1.39	94.06	2.938	2.112	86.57
AN18N	3.8	1.6	95.32	0.027	0.056	86.41	2.02	2.08	80.95	1.108	7.836	72.04
AT16P	2.25	0.25	98.16	0.05	0.113	61.27	3.02	2.81	95.23	1.711	8.189	90.51
AT17N	2.34	0.26	97.04	0.149	0.047	91.05	2.37	2.29	85.67	1.519	4.408	65.44
GF17N	0.43	0.48	97.5	0.072	0.076	86.03	-	-	-	0.119	18.541	90.51
GF18N	8.04	0.51	98.32	0.017	0.036	79.7	0.06	0.57	77.16	-	-	-
KB17N	0.02	9.79	93.64	0.142	0.087	90.4	1.49	9.07	78.12	-	-	-
MK16P	1.19	0.98	94.65	-	-	-	1.33	1.36	93.32	-	-	-
MK17N	0.14	0.53	99.06	0.07	0.045	84.84	-	-	-	2.546	4.491	90.51
MK18N	0.26	0.32	95.68	0.015	0.028	84.34	-	-	-	2.609	6.881	64.04
PW16P	6.39	0.97	98.43	0.028	0.032	89.43	2.83	5.14	94.49	0.972	1.195	83.69
PW17N	4.4	2.7	99.12	0.011	0.007	90.34	1.26	5.74	93.75	2.736	6.505	85.3
SK18N	8.63	4.98	95.27	0.026	0.041	84.24	5.33	5.34	88.78	0.242	1.951	69.97
SR18N	7.6	2.28	97.96	0.027	0.123	83.79	1.38	1.56	94.87	-	-	-

As an aid to interpreting the correlation structure between trials and investigating any genotype by trial interaction, the approaches of Kally et al. [9] as well as Cullis et al. [28] were followed. These authors use the dendrogram for cluster analysis and heat map representation of the genetic correlation matrix after fitting the FA models with the aim of grouping the trials into meaningful clusters that will be used for prediction and selection.

**Fig 3:**
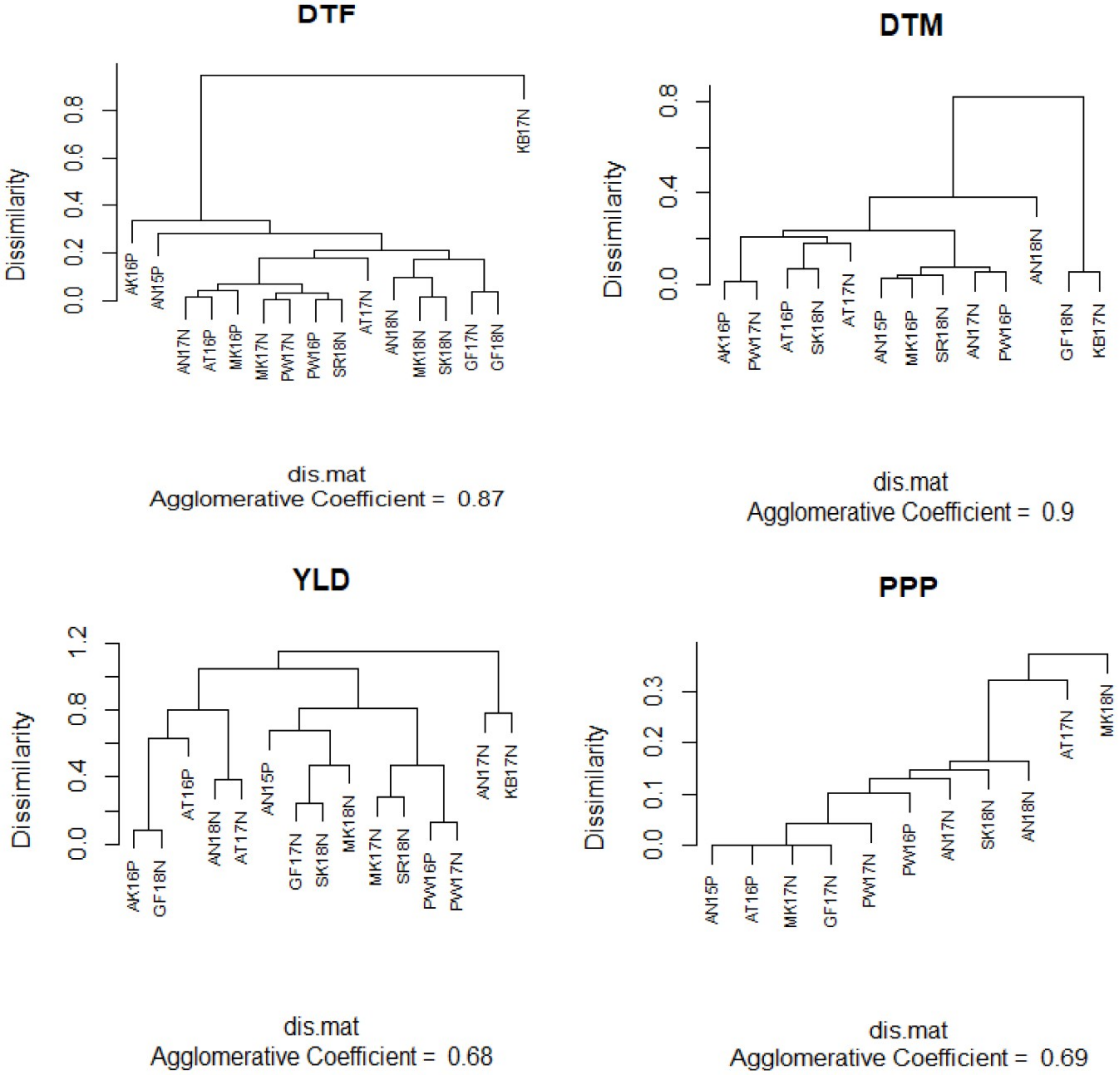
Dendrogram of the dissimilarity matrix from the final FA models fitted to the Yield (YLD), Plant per Pod (PPP), Day to flowering (DTF), and Day to Maturity (DTM).

In this paper, the cluster analysis using the dendrogram shown in Fig 3 was used to group trials based on genetic similarity. Based on Cullis et al.’s [28] suggestion on the dissimilarity cut-off (approximately below 0.6) that clusters are formed, the dendrogram (Fig 3) suggests possibly two clusters of trials for DTF and DTM, where one cluster is comprised of at most two trials, and only one cluster was identified for PPP. This shows that the genotype ranking is almost similar for all trials found within these formed clusters, and a different ranking of genotype for the trials found in different clusters. However, we can find about eight clusters of trials for YLD, and this implies that we would have different genotype rankings for a range of clusters of trials for this particular trait. In this regard, yield is a complex trait, which could potentially have high GEI effects, and here it requires an FA-4 model to adequately explain the GEI pattern. Genotype selection, therefore, was performed for each cluster using average BLUPs as a selection index, provided that the formed clusters are reasonably justified for making genotype selection independently for each of the clusters.

**Fig 4:**
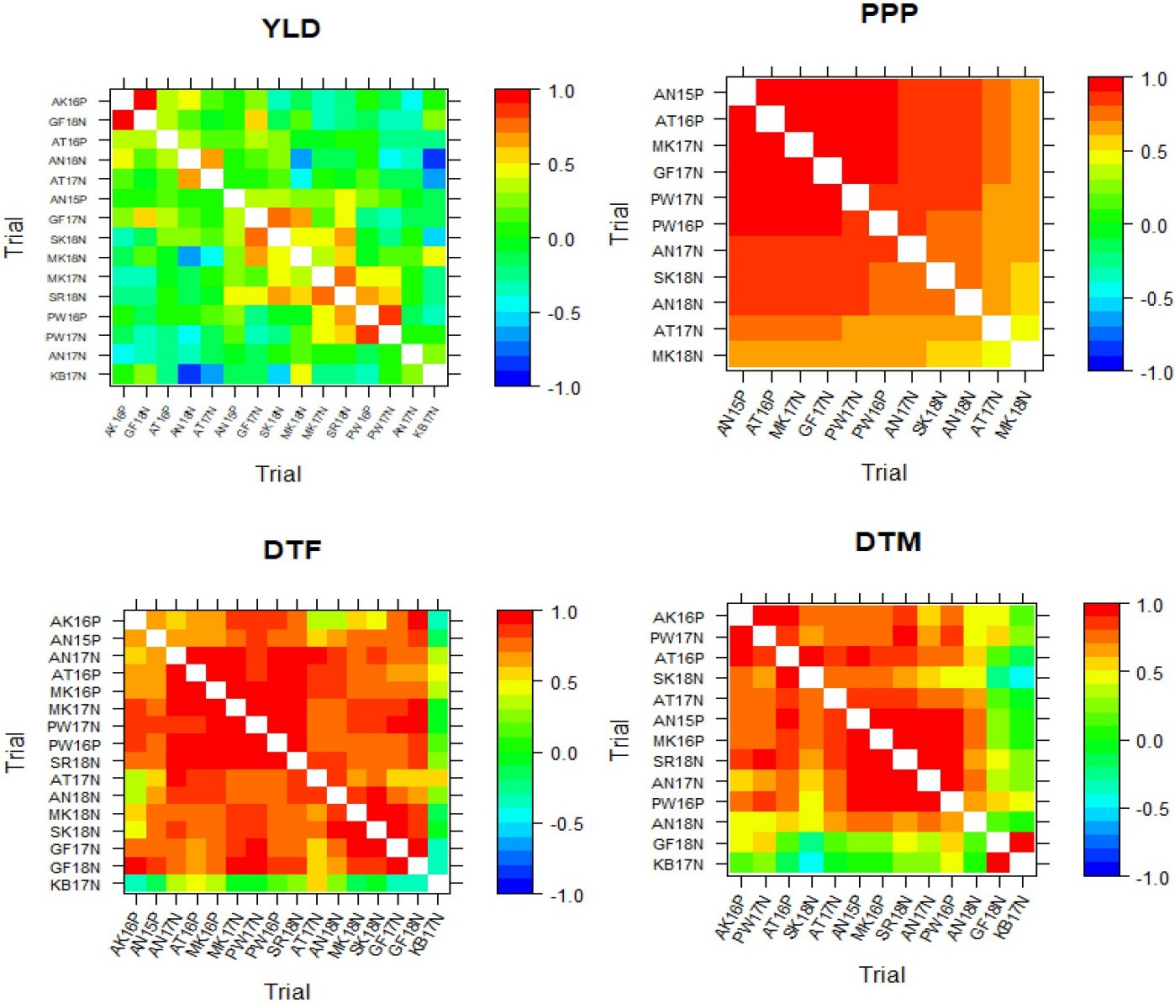
Heat map representation of the genetic correlation matrix from the final *fa* models fitted to the grain yield (YLD), plant per pod (PPP), day to flowering (DTF), and day to maturity (DTM).

In addition to the dendrogram, other typical summaries from the MET analysis include a heatmap of the genetic correlations between all trials for each trait. These are presented in Fig 4, which shows the different correlation patterns for each trait. From the heatmap, we can see most of the trials are highly correlated for the DTF, DTM, and PPP, and have a weak correlation for the YLD. This indicates that it is possible to carry out genotype selection through averaging of genotype means over nearly all trials for the DTF, DTM, and PPP. However, BLUPs for genotype means should not be averaged over trials for YLD since the genetic correlation is weak between most of the trails. There were also some trials that have a strong negative genetic correlation with most trials for YLD, such as trials AN18N and KB17N, suggesting that there may have been a reversal action in genotype rankings among these negatively correlated trials. Therefore, based on the dendrogram and heat-map (Figs 3 and 4) and the genetic variance as well from Table 5, we considered eight clusters of trials (C1, C2…C8) for YLD, where KB17N was in C1; AN18N and AT17N in C2; AN17N in C3; AK16P and GF18N in C4; AT16P in C5; AN15P in C6; GF17N, MK18N, and SK18N in C7; PW16P, PW17N, and MK17N in C8. Similarly, two clusters were considered for DTF and DTM, where KB17N was placed in one cluster for DTF, and GF18N and KB17N were placed in one cluster for DTM. We found only one cluster for PPP. In this paper, we used an average of BLUPs as a selection index to choose superior and stable varieties through ranking average BLUPs within clusters and assessing the stability across clusters.

Table 6 presents BLUPs for genotype means for all the traits across clusters of trials. With the exception of a trial conducted at Kobo, we found a cultivar, DAP 292, with a good performance for both days to flowering and days to maturity, but for yield only across four clusters of trials, C2, C3, C5 and C7. The yield advantage of this cultivar relative to the check across these clusters’ ranges from 10 to 32%. We also found that the cultivar DAP 292 had somehow a comparable yield with the check across the rest of the clusters. Conversely, the advantage of the number of pods per plant (PPP) of the best cultivar, DAB 292, over the check variety is the reverse.

**Table 6:** BLUPs for genotype means across clusters avarged over correlated trials.

	YLD								DTF		DTM		PPP
Genotype	CL1	CL2	CL3	CL4	CL5	CL6	CL7	CL8	C1	C2	C1	C2	C1
**DAB 292**	**1.75**	**2.80**	**4.16**	**1.07**	**2.23**	**2.55**	**1.72**	**1.84**	**44.7**	**34.4**	**77.1**	**77.6**	**11.2**
DAB 247	1.30	2.78	3.70	1.02	2.21	2.56	1.61	2.22	44.8	35.0	77.0	78.9	11.1
DAB 288	1.47	2.73	3.29	1.06	2.30	2.51	1.70	2.20	44.8	35.0	76.7	79.4	11.5
DAB 251	1.48	2.73	3.59	1.02	2.52	2.53	1.60	2.12	44.7	36.4	75.9	80.1	13.6
DAB 237	1.95	2.63	3.81	0.91	2.13	2.59	1.69	2.15	44.7	34.7	77.3	77.9	13.1
DAB 220	1.29	2.62	3.67	0.75	2.03	2.51	1.43	2.16	44.7	35.0	77.7	79.4	11.4
DAB 320	1.71	2.57	3.83	0.95	1.97	2.41	1.49	1.90	45.2	37.7	76.1	79.7	12.6
DAB 396	1.82	2.57	3.19	1.19	2.45	2.52	1.79	1.88	44.8	35.1	76.7	78.0	12.3
NUA 94	1.88	2.53	3.62	1.06	2.24	2.46	1.54	1.96	44.5	38.2	76.5	80.8	11.6
DAB 298	2.05	2.49	3.62	0.99	2.15	2.52	1.71	1.92	44.7	35.2	78.2	79.4	11.0
DAB 283	1.87	2.49	3.62	0.94	2.34	2.18	1.47	1.78	44.8	35.3	76.4	77.5	13.5
NUA 99	2.03	2.40	3.76	0.96	2.11	2.42	1.46	2.00	44.7	38.1	76.4	81.3	11.8
DAB 245	1.76	2.33	3.96	0.93	2.24	2.50	1.77	2.20	44.8	35.1	76.2	78.5	10.2
DAB 278	1.77	2.29	3.34	1.07	2.41	2.59	1.77	2.05	44.7	35.2	75.8	78.3	11.5
**GLP 2(Check)**	**2.60**	**2.21**	**3.78**	**1.12**	**2.02**	**2.43**	**1.30**	**1.98**	**44.8**	**40.1**	**77.5**	**82.2**	**13.8**
DAB 265	2.27	2.17	4.02	0.92	1.89	2.54	1.72	2.13	44.7	35.2	77.0	79.4	13.4
DAB 259	2.08	2.08	4.01	0.93	2.19	2.50	1.84	2.16	44.7	35.0	76.4	77.6	12.6
DAB 241	2.38	2.06	3.64	1.00	2.27	2.44	1.56	2.04	44.8	35.2	76.3	77.9	10.9

YLD: Grain yield; PPP: Pod per plant; DTF: Day to flowering; DTM: Day to maturity; CL1, CL2…C8 formed clusters for each trait.

## Discussion

Crop breeding programs’ main goal is to develop, evaluate and identify improved varieties that can result in significant yield improvement that can ultimately translate into economic gains. This needs to be accompanied by providing reliable information on the performance of the new varieties relative to existing ‘standard’ varieties. Many crop breeding programs conduct multi-environment trials to develop improved varieties and go through a series of variety evaluation stages. The term "genetic gain," which is frequently defined as the breeding terms of the breeder’s equation, has been used to describe genetic improvement or breeding progress that has been used as a key performance indicator (KPI) for assessing the overall breeding process. Genetic gain can be increased through increasing heritability, and much progress cannot be achieved from selections, if heritability is low. The best varieties can be chosen for upcoming crossings if heritability is high, further enhancing the breeding program’s genetic gain. The improvement of accuracy (heritability) in MET data analyses can be achieved using efficient statistical methods (Using sound experimental design and appropriate statistical analyses). This can be accomplished through modeling genetic effects (Genotype by environment (GxE) effects) and non-genetic effects (error variance heterogeneity within and between trials).

Mixed models work well for modeling both genetic and non-genetic effects in extended analyses of MET data (including incomplete MET data). We demonstrated the benefit of spatial analysis for the component associated with non-genetic effect by modeling spatial variation, using the methods popularized by Gilmour et al. [18], focusing on three scales; global, extraneous, and local trends. In comparison with non-spatial (conventional) analysis, spatial analysis provided relatively better improvements in MET data analysis. It is common practice in experiment design to control non-genetic variability by blocking, which is based on observable factors like soil type and topography. However, several potential inputs are unknown or unmeasured, which increased the variance of non-genetic effects. This emphasizes the significance of using spatial analysis for each trial in order to increase the precision and accuracy of variety evaluation.

Spatial analysis is essential for field trials in Ethiopia, where soil and environment are highly variable, even within very small plots, as noted in [29]. If this spatial variation is ignored, variety valuation results will be biased and ineffectual. Our study revealed that spatial analysis had a role in increased the heritability in the majority of trials. As a result, there is potential for a remarkable enhancement in the precision and accuracy of variety evolution. This improvement can be attributed to spatial analysis’ capability to effectively capture non-genetic variation linked to the variability in agricultural field plot [17, 28]. The research conducted in varietal field trials on common bean [30], maize [31], and sorghum [32] consistently reveals compelling evidence of the profound benefits of spatial analysis. These studies, with a common focus on the method of analysis, highlight the advantages of incorporating spatial analysis techniques to disentangle non-genetic effects that may even be intertwined with the true genetic effect.

We showed that the analysis of MET data was significantly improved by the FA models developed by Smith et al. [33], which were used to model genetic effects. More importantly, modeling genetic effects using FA models in conjunction with spatial models for non-genetic effects significantly improves the analysis of the MET data set. This was also demonstrated in related studies by Cullis et al. [28] and Kelly et al. [15], The FA models have been found to be useful not only for accurately estimating/predicting genetic effects, but also for estimating their variance and performing graphical analysis. Correlated environments can be identified using estimated genetic variance, and breeders can select genotypes using BLUPs averaged across correlated environments.

The majority of studies use a two-stage approach to analyze GxE effects, estimating genotype by environment from individual trial fixed effect tables. Peixouto et al. [34] and Sinebo and Taye [35] conducted the GxE analysis through fixing genotype as random for both separate and combined analysis. The genotype by environment means table were BLUPs estimated from the individual trial data analysis. Smith et al. [33] criticized this approach, claiming that it results in a double-shrinkage in the estimation of G×E effects and that it must be unshrunk back to fixed-effect estimates.

Our analysis for GxE effects uses a one-stage approach that addresses the issue of double-shrinkage in the estimation of GxE effects by simultaneously estimating models for residual effects and GxE effects. Gogel et al. [13] reported that the one-stage analysis method outperforms the two-stage method and is the gold standard for analyzing MET data. The two-stage analysis, which approximates one-stage analysis, is a more practical strategy, especially when we are dealing with an incomplete data problem and a computational facility. Our findings show that both the spatial and factor analytic models based on a one-stage approach improved the analysis of common bean multi-environment trial data significantly, and this should be implemented in the beans breeding program as a routine stand for the analysis of MET data.

The utilization of spatial and FA models for the analysis of MET data presents a promising avenue for enhancing the precision and accuracy of genotype evaluation. By integrating these models into the analysis, non-genetic variations that are intricately linked to agricultural field experiments can be effectively captured. This allows for a more comprehensive exploration and exploitation of the valuable information stored within the MET dataset [28, 29].

Some studies conducted MET data analysis, employing Factorial Analysis (FA) models, to evaluate the performance of sorghum and wheat genotypes [30, 36]. These studies employed the BLUPs averaged across multiple trials, to provide a robust assessment of the genotypes’ performance. In our study, however, the performance of each genotype is evaluated using BLUPs, taking into account the correlation patterns among trials for each trait observed in the heat map analysis. As noted by Kelly et al. [9], averaging BLUPs across trials is not recommended in the presence of a weak genetic correlation, as it may not accurately represent the true potential of the genotypes. Thus, caution is advised when it comes to averaging BLUPs over trials for YLD, as there is a weak genetic correlation observed among the majority of trials. Furthermore, certain trials exhibit a pronounced negative genetic correlation with the majority of trials for YLD. This observation implies that there might have been a reversal in the ranking of genotypes among these negatively correlated trials. Our study’s findings also showed a strong correlation across trials for DTF, DTM, and PPP, providing strong support for genotype selection through averaging genotype BLUPs across nearly all trials for these traits.

The genotype DAB 292 exhibited greater performance in the majority of clusters of trials for DTF, DTM, and YLD. However, the advantage of the number of pods per plant (PPP) of this genotype over the check variety is the reverse. The reason for the higher productivity but lower number of pods per plant for this genotype might be that it has higher number of seeds per pod. In such cases, recording the number of seeds per pod might be advantageous for conformation.

## Conclusion

The MET data is not always balanced and/or complete, and ANOVA-based techniques may not be suitable for its analysis. The linear mixed model provides a strong framework for dealing with imbalanced and/or incomplete data, as well as relaxing the ANOVA distributional assumptions surrounding the residual error. Under the linear mixed model, the spatial and FA models were found to be an efficient method of data analysis for this study. The multiplicative spatial mixed (Spatial+FA) model analysis provides significant improvement in the MET data analysis results, and this is demonstrated with the evidence of increased heritability. The improvement in the analysis is due to the fact that each individual trial is spatially analyzed and the genetic effects are modeled using FA models. Thus, the analysis of multi-environment trial data through the use of more efficient statistical models (such as multiplicative spatial mixed models) can provide a more robust platform for evaluating common bean varieties with greater confidence in selecting superior varieties across a range of environments. Hence, scaling up the use of such an efficient analysis method is indispensable for enhancing the selection of superior varieties.

## Supporting information

S1 File Grain yield and yield related data. (TXT)
